# Improving Road Traffic Forecasting Using Air Pollution and Atmospheric Data: Experiments Based on LSTM Recurrent Neural Networks

**DOI:** 10.3390/s20133749

**Published:** 2020-07-04

**Authors:** Faraz Malik Awan, Roberto Minerva, Noel Crespi

**Affiliations:** Telecom SudParis, Institut Polytechnique de Paris, CNRS UMR5157, 91000 Evry, France; roberto.minerva@telecom-sudparis.eu (R.M.); noel.crespi@mines-telecom.fr (N.C.)

**Keywords:** air pollution, atmospheric data, deep learning, IoT, LSTM, machine learning, RNN, Sensors, smart cities, traffic flow, traffic forecasting

## Abstract

Traffic flow forecasting is one of the most important use cases related to smart cities. In addition to assisting traffic management authorities, traffic forecasting can help drivers to choose the best path to their destinations. Accurate traffic forecasting is a basic requirement for traffic management. We propose a traffic forecasting approach that utilizes air pollution and atmospheric parameters. Air pollution levels are often associated with traffic intensity, and much work is already available in which air pollution has been predicted using road traffic. However, to the best of our knowledge, an attempt to improve forecasting road traffic using air pollution and atmospheric parameters is not yet available in the literature. In our preliminary experiments, we found out the relation between traffic intensity, air pollution, and atmospheric parameters. Therefore, we believe that addition of air pollutants and atmospheric parameters can improve the traffic forecasting. Our method uses air pollution gases, including $CO,NO,NO_{2},NO_{x},$ and $O_{3}$. We chose these gases because they are associated with road traffic. Some atmospheric parameters, including pressure, temperature, wind direction, and wind speed have also been considered, as these parameters can play an important role in the dispersion of the above-mentioned gases. Data related to traffic flow, air pollution, and the atmosphere were collected from the open data portal of Madrid, Spain. The long short-term memory (LSTM) recurrent neural network (RNN) was used in this paper to perform traffic forecasting.

## 1. Introduction

### 1.1. Motivation

Vehicular traffic management is a major issue in cities and metropolitanareas [1]. Traffic has a relevant impact on different aspects of daily life, from time spent in traffic jams to higher level of pollution produced, from gas and resources consumption to infrastructural investments and maintenance of road and transportation systems [2]. Traffic management and optimization are essential parts in every smart city platform. Smart mobility is one of the most important services of smart city platform. It has a direct impact on the quality of life of citizens and on the ability of the city to support the exchange of people and goods within the urban environment. Traffic regulation and orchestration are key components. With a city’s large number of vehicles, problems related to traffic are critical for the effective functioning of the city and the health of its citizens. Traffic congestion is a major problem, especially when it is associated with an increasing number of vehicles in use (e.g., in cities with inadequate public transportation). It leads to environmental, social, and economic issues [3]. The timely prediction of traffic flow can be helpful to avoid congestion, as drivers can choose the most comfortable and less congested path to reach their destination, or modify their time schedule for their journey in order to compensate for the expected time of arrival caused by the traffic. Road traffic forecasting is defined as the estimation or prediction of the traffic flow in the (near) future. Another aspect of traffic levels in cities is car and truck generated air pollution. Many cities suffer from air pollution. Increasing traffic emissions is one of the major contributors to urban air pollution [4]. According to the World Health Organization (WHO) [5], a large portion of air pollution is contributed by the transport sector. These two phenomena are linked, and many cities are tackling this problem by deploying sensors for measuring traffic intensity and air quality. Air pollution generated by traffic depends on several factors, ranging from the types of vehicles (gasoline, diesel, electric), to the level of congestion and the time spent in traffic jams, the atmospheric or geographical characteristics of the environment, and many more.

**Figure 1:**
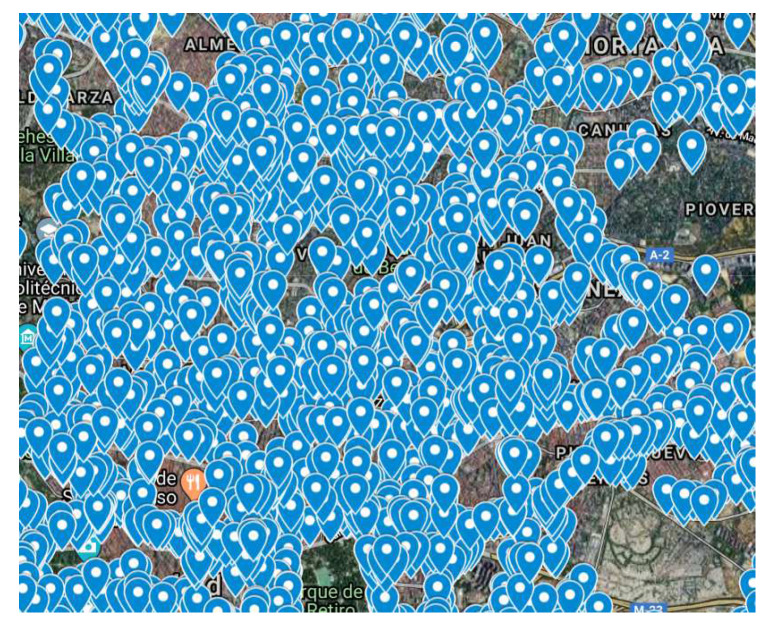
Traffic intensity sensors in Madrid.

**Figure 2:**
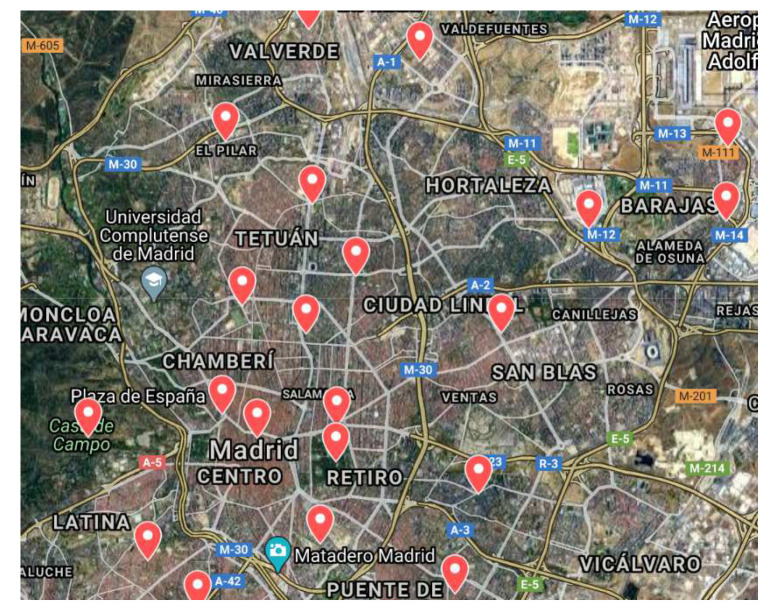
Air pollution sensors in Madrid.

**Figure 3:**
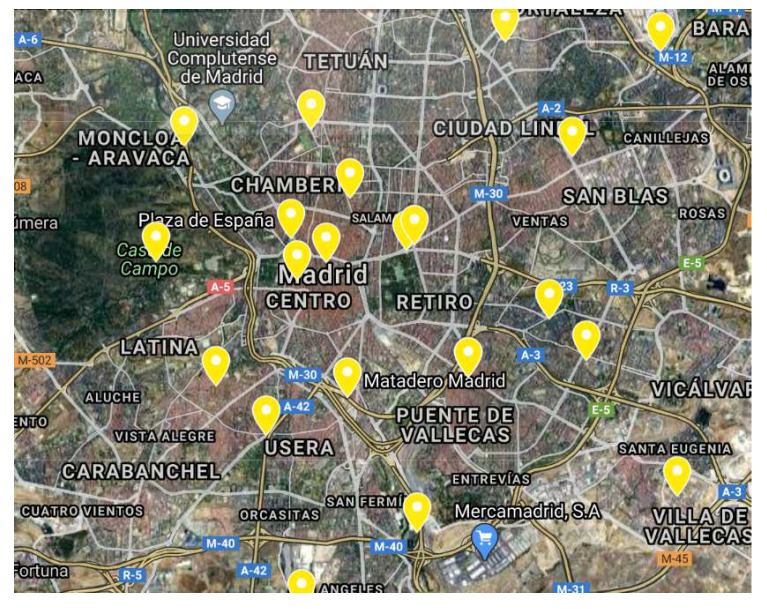
Weather stations in Madrid.

**Figure 4:**
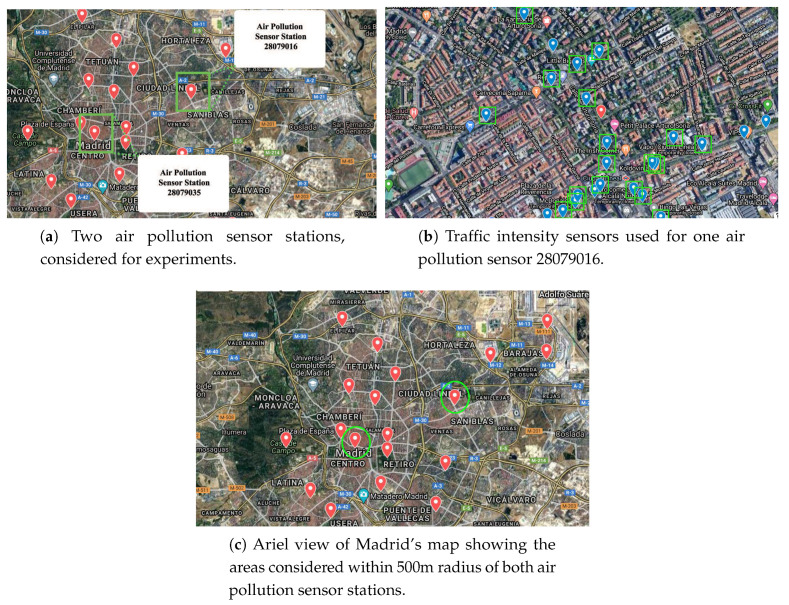
Considered air pollution sensor stations, traffic intensity sensors, and areas in Madrid.

A large networks of sensors have already been deployed in several cities (e.g., Madrid, Santander, and Barcelona in Spain, Singapore, Seoul, Copenhagen). Data generated by these sensors are very useful for forecasting. For example, around 4000 traffic intensity sensors are deployed in Madrid, Spain (Figure 1) [6]. These sensors provide information about the number of vehicles passing per hour (actually every 15 minutes). Similarly, there are 24 stations measuring air pollution (Figure 2) and 26 stations collecting atmospheric data such as local temperature, pressure, wind speed, and wind direction (Figure 3). Madrid’s data, then, offer the possibility to further analyze the correlations between traffic intensity, levels of pollution, and meteorological condition. Figure 1, Figure 2 and Figure 3 show that traffic intensity sensors are greater in number as compared to air pollution sensors. Air pollution sensor data are not so granular as the traffic intensity ones. Therefore, in our experiments, we chose traffic sensors in close proximity (upto 500 m) (Figure 4c) to air pollution sensors and, vice versa, we selected air pollution sensor stations close to big roads or crossroads. Air pollutants such a $CO,NO,NO_{2},NO_{x},$ and $O_{3}$ are associated with road traffic [7,8,9]. The combination of large quantities of curated data with machine/deep learning models can provide useful insights for the correlation of traffic with air pollution. Many studies demonstrate how data about traffic flow can be used to predict air pollution. For example, Batterman et al. [10] used a dispersion model, called the Research Line Source (R-LINE) model, and emission inventory to predict the air pollutants PM2.5 and $NO_{x}$. Ly et al. [11] predicted the concentration of $NO_{2}$ and CO by using multisensor devices data and weather data, including temperature, relative humidity, and absolute humidity. In this work, they used the data of an Italian city (unnamed city) between March 2004 and February 2005. Similarly, Lana et al. [12] used a Random Forest regression model to predict the air pollution level with respect to road traffic utilizing open data from Madrid for the year 2015. Russo et al. [13] used atmospheric data, including temperature, wind direction, wind intensity, along with other air pollutants, including $NO_{2}$, NO, and CO as input variables to neural network to forecast the concentration of PM10. However, in their experiments, they did not take traffic intensity into account. Brunello et al. [14] investigated temporal information management to assess the relationships between air pollutants, including $NO_{2},NO_{x},$ and PM2.5, and road traffic. In all of these studies, thanks to the direct link between road traffic and air pollutants, road traffic was used to predict air pollution. Air pollution and traffic intensity data are collected as time-series of values and are generally made available for analysis and study. However, to the best of our knowledge, there has not yet been an attempt to use air pollution to improve the traffic forecasting. Traffic intensity is a major contributor to air pollution. The presence of certain pollutants in the air is most likely determined (or largely contributed) by vehicle traffic. Being able to correlate the actual level of these pollutants, on a timely basis for an area close to an air pollution station, to the expected level of traffic in the same area can be of help in better predicting the traffic intensity. Hypothetically, if the only source of pollution was car traffic, a strong correlation between the air pollution level and the intensity of traffic could be drawn. Cities and urban conglomerates are complex systems and there are other major contributors to air pollutions (home heating, factories and transformation implants, and others). Besides this, also meteorological condition can influence the air quality, e.g., strong winds can spread and disseminate pollutants in large areas making it more difficult to find strong correlations between traffic, air pollution and other contributors. In spite of the complexity of these causal relations, Madrid offers an impressive wealth of data for approaching and further study the correlation between traffic intensity and air pollution. The analysis considers the current level of pollution in a specific area at a specific time interval “*t*” as an evidence of presence of traffic. This evidence is also reinforced by the ability to know the traffic intensity levels before the time “*t*”. Using these data could lead to a better prediction of the traffic intensity. Generally speaking, the approach of considering air pollution data as a means to predict traffic intensity can be undertaken in two ways: to use air pollution data together with traffic intensity data to improve the prediction of traffic intensity, or to use the air pollution data and numerical models to infer the expected traffic intensity. This paper evaluates the first option, while the second one is left for further study.

Cities are systems that attract peoples, goods and activities and their impact is not limited to the city limits, but extend to cities, towns, and villages in the surrounding area. According to a World Economic Forum report [15], people prefer living, staying, studying, and growing up in cities. In fact, big cities exert a strong attraction effect and have a considerable impact on very large areas. The traffic and pollution issues involved may therefore be better analyzed if the extended areas are considered. Sometimes, air quality measurements are also assessed in decentralized areas. Thanks to the availability of several open datasets, it is possible to investigate the correlation between air pollution and traffic intensity that may have contributed to the level of pollution in large monitored areas. This information will in turn offer the possibility to focus on air quality analysis and to correlate it to the expected traffic intensity. This paper investigates this possibility, starting from a highly-sensed and populated area (Madrid and its surrounding area). In Madrid’s data portal, datasets related to air pollution and atmospheric data are available timely each hour. On the other hand, data for traffic flow is updated every 15 minutes. Historic data of traffic flow, air pollution, and atmospheric variables for each month is made available at the end of the month. One expected outcome of this work is to validate (or reject) the usage of current air pollution measurements and levels combined with atmospheric data to improve the prediction of the traffic intensity levels.

Traffic intensity is the major cause of the pollution problem. So not surprising, measuring or using the resultant levels of pollution generated can be a means to understand how many vehicles may be present. Pant et al. [16] performed an analysis to characterize the traffic-related PM emissions in a tunnel environment. For this purpose, they chose 545 meters long, one of the major tunnels in Birmingham, called A38 Queensway Tunnel. Around 25,000 vehicles travel through this tunnel daily. They deployed the PM sensors at the distance of 1.5 m on emergency layby. A similar experiment can be done with different number of vehicles to observe the volume of the pollution produced. A set of vehicles operating for a specific period of time in the same area will produce a very similar quantity of pollutants (imagine 100 cars in a closed environment, they will produce the same amount of pollutants when operating for the same period of time). Measuring the levels of pollutants over time may create a dataset usable to predict level of pollution as well as from the pollution levels to determine how many cars were contributing. Hypothetically, measuring the level of pollution at a certain instant may allow to determine how many cars were operating. In the real-world, things are more dynamic, for instance:

- the concentration of pollutants is greater close to big roads [17] (this is also why we tried to consider traffic intensity sensors close to the pollution sensors).
- the set of vehicles may be dynamic in composition (more diesel, more electric, and so on) during the days.
- the pollution level generated can be impacted by the meteorological condition.

However, the traffic in a city shows patterns and in spite of the dynamic of the composition/aggregation of vehicles producing pollutants, there are patterns also in how people use the cars (e.g., similar number of commuters in peak hours of traffic). These patterns are also well-known by users, they, in fact, expect to have different traffic condition during the day and the week (with large differences between working days and week-ends). Over a long period of time, these patterns repeat and the levels of pollution can be considered as signatures of traffic intensity. The hypothesis to verify is if the levels of pollution may correspond on the average to certain levels of traffic and if these measurements of pollution can be used to improve the traffic predictions. Having time-series of the pollution signatures together with time-series of traffic intensity will allow to better predict the traffic intensity.

The objective is also to determine if such an approach is practical and if it can give useful and improved results over an analysis that considers only the traffic intensity time-series. Determining the relations between levels of pollution and traffic intensity may lead to important consequences such as: to better control the air quality in more parts of the city and still maintain the desired levels of monitoring of vehicular traffic situation; the reduction of the number of traffic sensors, which can lead to reduced maintenance costs that could go in favor of a more capillary environment management infrastructure; moving from specific sensing and monitoring to general-purpose sensing for large urban environments [18]; the integration and exploitation of other forms of environmental control (e.g., satellite data).

**Figure 5:**
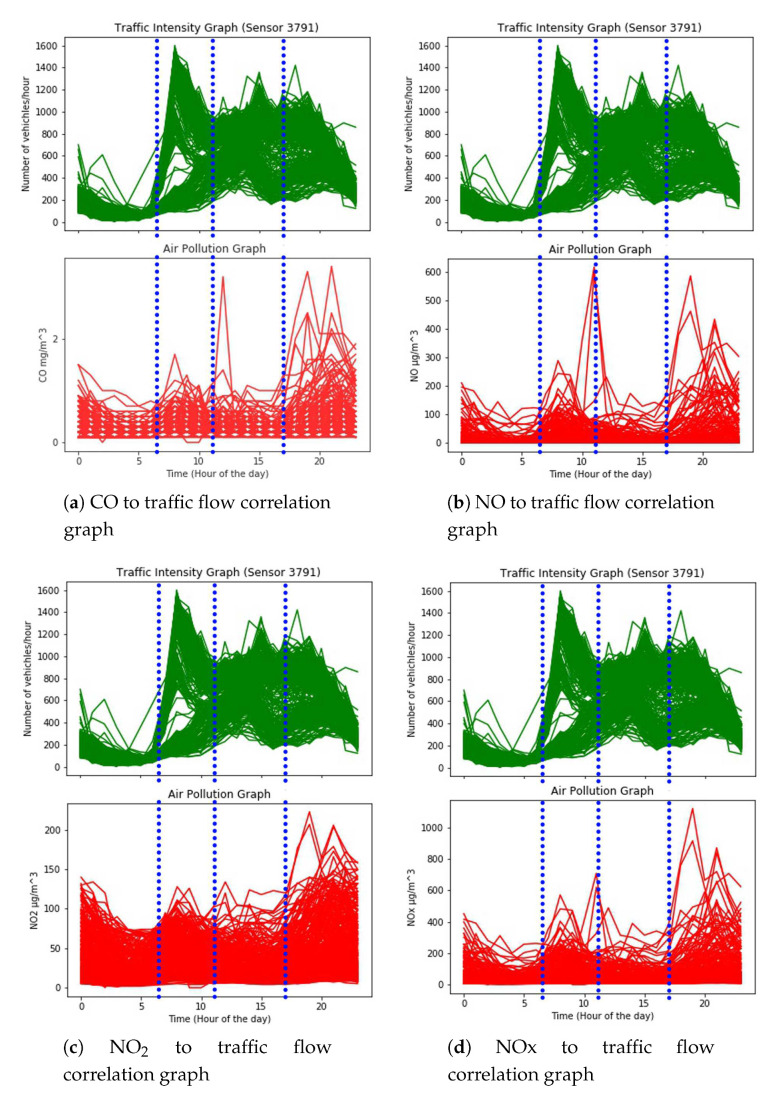
Correlation graphs of traffic flow and air pollutants with respect to each hour of the day.

**Figure 6:**
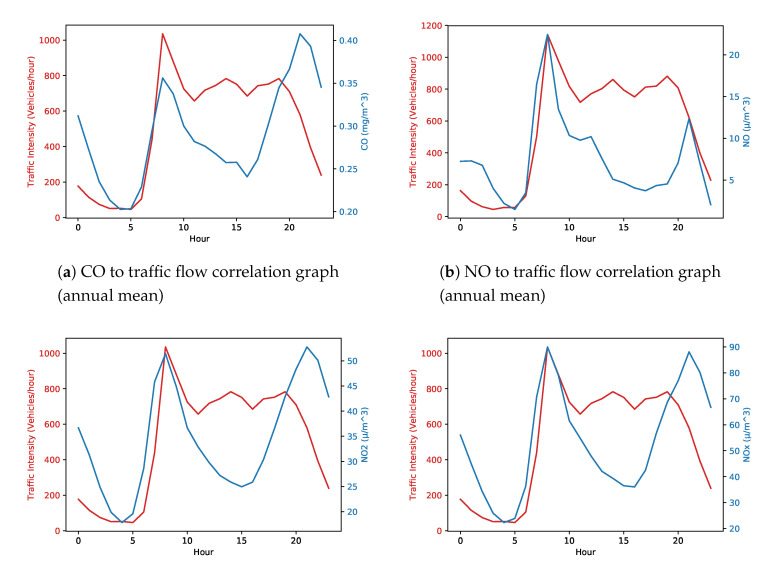
Correlation graphs of traffic flow and air pollutants with respect to each hour of the day (annual mean).

LSTM recurrent neural network is very popular for dealing with time-series data [19]. In the case at hand, the relationship between traffic intensity and pollution levels are aligned (see Section 3.1 and Figure 5 and Figure 6), other time the relationship is blurred by other factors (e.g., meteorological factor). Neural Network can be fruitfully used to capture the evident and the more hidden patterns. For instance, in a week period different patterns (working days versus week-end may show different courses). An adequate period of time for a repeated number of time (e.g., a weekly observation for a duration of a year of data) may disclose relevant correlations. Therefore, we adopted a long short-term memory (LSTM) recurrent neural network (RNN)-based approach which uses air pollutants, including $CO,NO,NO_{2},NO_{x},$ and $O_{3}$, along with some atmospheric variables including pressure, temperature, wind direction, and wind speed to improve road traffic forecasting in Madrid, Spain. The experiments presented in this paper are based on one year of data collected from Madrid’s open data source. Complete details about the dataset are provided in Section 4.

### 1.2. Contribution

With this paper, we have made the following contributions:

- We provide a detailed statistical analysis based on the relationship between air pollutants, atmospheric variables, and road traffic;
- To the best of our knowledge, this is the first attempt to use air pollutants in combination with atmospheric variables to improve traffic forecasting in a smart city;
- Our approach uses a well-known LSTM RNN for time-series traffic data forecasting; and
- We provide some proof of the validity of our approach and avenues for future work.

### 1.3. Organization

The paper is organized as follows. Section 2 offers a summary of the related work, and Section 3 explains the methodology. The dataset information and performance evaluation are provided in Section 4, and Section 5 concludes the paper and indicates promising directions for future work.

## 2. Related Work

In this section, we summarize the existing work on traffic forecasting available in the literature. Ji et al. [20] used a deep learning, LSTM RNN-based model exploiting long-term evolution (LTE) access data as an input to their model for the prediction of real-time speed of the traffic. Similarly, Wei et al. [21] proposed an AutoEncoder and LSTM-based method to predict traffic flow. They collected data from the Caltrans Performance Measurement System (PeMS) and considered only three features: (1) traffic flow, (2) occupancy, and (3) speed. Li et al. [22], in their paper, provide an overview of the machine learning approaches for short-term traffic forecasting. Ketabi et al. [23] provide a comparative analysis of multiple variant recurrent neural network and conventional methods for traffic density prediction. They used 40 day data, generated by 58 cameras in London, of the time slot between 9:30 AM and 6:30 PM. Their work considered two features: time and traffic density. Zhu et al. [24] used GPS information data to develop a traffic flow prediction model. Based on data clustering using historic GPS data, their artificial neural network-based prediction model utilized a weighted optimal path algorithm to predict short-term traffic flow. This prediction, based only on the departure time, was then used as input to an A-Dijkstra algorithm to find an optimal path.

Hou et al. [25] proposed a hybrid model that combines an autoregressive integrated moving average (ARIMA) algorithm and a wavelet neural network algorithm for short-term traffic prediction. Their experiment is based on a case study of the Wenhuadong/Tongyi intersection in Weihai City, and only considers weekdays. They collected data over three workdays, using the data from first two days for training and 3rd day’s data for testing. Time and traffic flow were the only two features considered. Similarly, Tang et al. [26] proposed a hybrid model, comprising denoising schemes and support-vector machines for traffic flow prediction. To conduct their experiments, they collected data from three traffic flow loop detectors deployed on a highway in Minneapolis, MN (USA). They considered five denoising methods (Empirical Mode Decomposition, Ensemble Empirical Mode Decomposition, Moving Average, Butterworth filter, and Wavelet) for performance evaluation purposes. Their data contained three features: volume, speed, and occupancy. Wang et al. [27] presented an integrated method, combining Group method of data handling (GMDH) and seasonal autoregressive integrated moving average (SARIMA), for traffic flow prediction in the Nanming district of Guiyang, Guizhou province, China. They collected data for five working days; data from the 1st four days were used for training while the last day’s data were used for testing. They used residue series as features and labels, respectively to train the model. Rajabzadeh et al. [28] proposed an hybrid approach for short-term road traffic prediction. Based on stochastic differential equations, their approach ultimately improves the short-term prediction. They divided their approach into two steps: (1) a Hull-White model implementation to obtain a prediction model from previous days and (2) the implementation of an extended Vasicek model in order to model a difference between predictions and observations. Two datasets were used: one from a highway in Tehran, and the other an open dataset of PeMS time and traffic volume as inputs. Goudarzi et al. [29] proposed an approach based on self-organizing vehicular network to predict traffic flow. They used a probabilistic generative neural network technique, called deep belief neural networks, to predict traffic flow. Data generated by road side units (RSUs) were used for experiments, with traffic volume and time as inputs. Abadi et al. [30] used traffic flow series that indicate the trends in traffic flow; wavelet decomposition provided basis series and deviation series from the traffic flow data. In addition, local weighted partial least squares and Kalman filtering were used to predict the basis series. One day’s data (8:00 AM to 8:00 PM) from the website of the ministry of communication of Taiwan were used for their experiments. Zhang et al. [31] used atmospheric data (average wind speed, temperature, ice fog, freezing fog, smoke) as input to gated recurrent neural network to predict the traffic flow. Rey del Castillo [6] presented an analysis on Madrid’s traffic. In this work, short-term indicators of traffic evolution have been produced. Similarly, Lagunas [32] used different machine learning algorithms, including K-means, K-nearest neighbors, and Decision Tree, combined with traffic data, weather data, and data related to events in Madrid to predict the traffic congestion in an area.

The majority of the above-mentioned works used traffic intensity and time in order to forecast traffic. However, we believe that some other parameters like atmospheric conditions can effect the traffic flow which have not been considered in above-mentioned works. Tsirigotis et al. [33] considered only rainfall, along with traffic volume and speed to forecast the traffic. Similarly, Xu et al. [34] considered temperature and humidity, along with taxi trajectory data to forecast traffic flow. They took travel time, pick-up & drop time, and distance into account to forecast traffic flow. Only one month’s data (01 January 2015 to 31 January 2015) were considered. We believe, traffic pattern can vary in different days and months. For example, we might observe different traffic pattern during weekends. Similarly, according to a case study in Copenhagen, Denmark, 80% journeys are made on foot in city center and 14% are made by bicycle in summer [35]. On the other hand, traffic forecasting based-on taxi trajectory might have other flaws too. For example, road lines leading to airports might have heavy traffic flow as compared to other lines in surrounding areas. Traffic forecasting for surrounding areas, based on taxi traveling in the lines with heavy traffic flow might result an inaccurate forecasting. In this paper, we are introducing the use of air pollutants and atmospheric parameters (pressure, temperature, wind direction, and wind speed) to forecast traffic. These are the two motivations for using atmospheric parameters: they influence the level of air pollutants in the air, and they also can influence the human behavior. For example, Badii et al. [36] used weather conditions, including temperature, humidity, and rainfall to predict the availability of parking spots inside parking garages, given the fact that depending on the weather condition, people’s choice of parking may vary. For example, in thunderstorm, people will prefer indoor parking. Similarly, on different occasions, people may prefer to use public transport which may affect the occupancy of parking lots.

## 3. Methodology

In this section, we describe the methodology for forecasting traffic flow using traffic intensity values. A first step was to use traffic intensity data combined with air pollution and atmospheric data in order to forecast the traffic. We correlate traffic intensity data to air pollution and atmospheric variables (as we also want to study the relationship between traffic and pollution). As described earlier, air pollutants are often linked to the road traffic levels. Using that link, we propose to use air pollutants and atmospheric variables to forecast the traffic flow. In the second step, we used only time-stamped traffic intensity data, excluding air pollutants and atmospheric data, to forecast the traffic flow. The results produced from step one and step two were then compared to observe how air pollution and atmospheric data, combined with traffic intensity data, could be used to forecast traffic flow. Our experiments were organized into two categories: (1) statistical analysis and (2) traffic forecasting using LSTM RNN. For our experiments, we used open data, collected by the city of Madrid, Spain [37]. The first category of experiments was instrumental for analyzing the quality of available data and to identify macroscopic properties of the data sets.

### 3.1. Statistical Analysis

**Figure 7:**
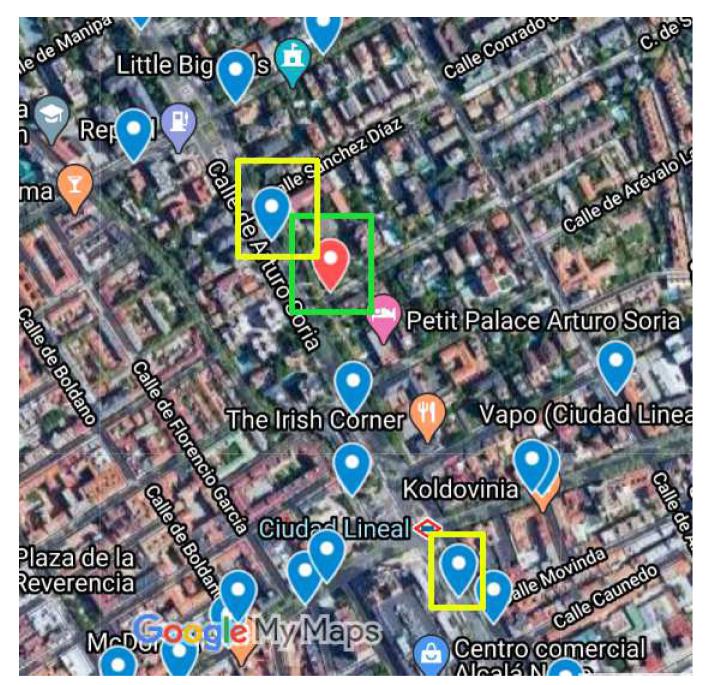
Considered air pollution station (highlighted by the green rectangle) and traffic flow sensors (highlighted by the yellow rectangles).

As the initial step, we chose one of the air pollution measuring stations and selected two traffic flow sensors at different distances (Figure 7). We collected hourly data from 01 January 2019 to 31 December 2019. This data contained the number of vehicles per hour that passed the sensors, and the air pollutants (CO,NO,NO2,NOx, and O3) levels. Subsequently, we used the accumulated data in order to have an initial view on the possible correlations and to determine a set of parameters that could have an impact on the correlation. We plotted the data on graphs in order to observe the traffic flow patterns with respect to air pollution, as shown in Figure 5. Figure 5 represent the hourly graph of traffic flow measures of one of the selected traffic flow sensors with respect to air pollutants CO, NO, $NO_{2}$, and $NO_{x}$. These graphs represent the values of each hour of each day of the year 2019. The graphs in green represent the traffic intensity while the corresponding graphs in red represent the air pollutant levels. In these graphs, blue dotted lines divide the graphs into four time intervals. During the first 2 intervals, all the measured air pollutants follow the traffic flow trend, with few exceptions. In the first interval, the pollutant levels decrease when the traffic is decreasing. Similarly, during the second interval, the pollutant levels increase when the traffic is increasing. A similar pattern can be seen during the fourth interval. However, during the 3rd interval, the pollutants do not seem to be following the traffic flow pattern. To investigate this phenomenon, we studied air pollution dispersion aspects and considered wind speed as one of the factors in air pollution dispersion [38].

**Figure 8:**
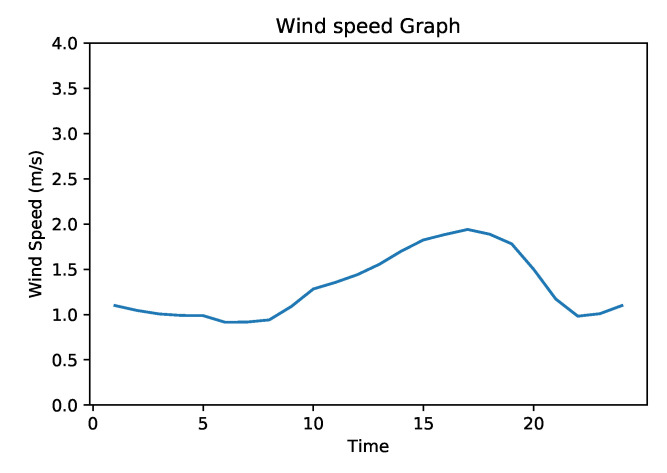
Average annual wind speed.

Hence, as a further verification, we plotted a graph representing the average annual wind speed for each hour (Figure 8), which reveals that wind speed is constantly increasing during the time interval when air pollution does not follow the traffic flow pattern. Given the air pollution dispersion values and the available data, we consider that wind speed is one of the factors that influence air pollution dispersion. As mentioned above, we noticed from statistical analysis that there are similarities in the growth of traffic and the growth of pollution during the morning, and there is a shift in the growth of traffic and the growth of pollution during the evening. In the mid of the day, the correlation is more difficult to capture. This is why we used RNN in order to determine some correlations beyond the statistical ones. The same algorithm using only traffic intensity data and using traffic intensity + meteorological + pollution data show different levels of precision in favor of the analysis that considers more contextual information (a comparative analysis is provided in the Section 4.2). Figure 5 presents the correlation between air pollutants and traffic intensity with respect to each hour of each day of the year. However, in order to provide more insights related to correlation, we have plotted an annual mean graphs for all the considered air pollutants (Figure 6). Phase shift can be seen in Figure 6 too, however, phase shift in Figure 6 is different than that of in Figure 5 because of average annual values.

### 3.2. Linear Interpolation

Missing values from the data is another major issue when dealing with time-series data. Even though the available open data of the city of Madrid is well maintained, minor glitches in sensors are almost inevitable. Sensors may go offline because of technical issues, or there is a possibility that received data could not be stored on a server. While conducting our initial data analysis, we observed that some of the traffic flow sensors had missing values for some timestamps. Though these missed values were not numerous, it was necessary to fill the gap because we were dealing with time-series data. In order to deal with this issue, we used a well-known method, linear interpolation. Linear interpolation is a popular technique to fill the missing values in a dataset [39]. This technique seeks to identify timestamps that are similar to those that are missing their values, and fills each missing value with an average value [40]. Linear interpolation states that there is a constant gradient in the rate of change between one sample point and the next point. Considering this assumption, if the amplitude of the $i^{th}$ point is $x_{i}$ and the amplitude of the $i+1^{th}$ point is $x_{i+1}$, then keeping the constant gradient, the $j^{t}h$ point between $x_{i}$ and $x_{i+1}$ can be calculated as follows [41]:

(1) $$\frac{x_{i+1}-x_{i}}{(i+1)-i}=\frac{x_{j}-x_{i}}{j-i}$$

or

(2) $$x_{j}=\left(j-i\right)\left(x_{j}-x_{i}\right)+x_{i}$$

### 3.3. Traffic Forecasting Using LSTM Recurrent Neural Network

**Figure 9:**
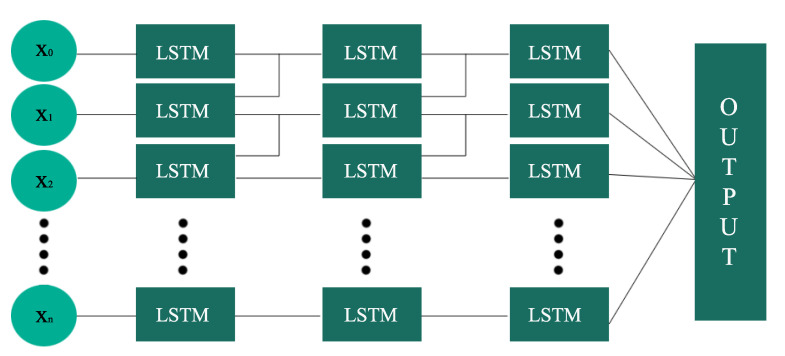
LSTM Recurrent Neural Network Architecture.

When dealing with time-series data or spatial temporal reasoning, the LSTM RNN is considered one of the best options. As shown in Figure 9, unlike traditional neural networks, the LSTM RNN has memory units instead of neurons. With traditional fully connected neural networks, there is a full connection between the neurons of two adjacent layers. However, there is no connection between the neurons within the same layer. This lack of connection in traditional neural networks could create problems, and may likely cause total failure in terms of spatial temporal reasoning [42]. In RNNs, a hidden unit (memory unit) receives the feedback. This feedback goes from previous state to the current state. We used $timestamp,day\_of\_the\_week,CO,NO,NO_{2},NO_{x},O_{3},pressure,temperature,wind\_direction,wind\_speed,$ and traffic_flow as the features for our RNN. If we denote the input for the model as $x=(x_{1},x_{2},x_{3},...,x_{T})$ and the output as $y=(y_{1},y_{2},y_{3},\ldots ,y_{T})$, with the *T* in *x* and *y* is the prediction time, the traffic flow prediction at time *t* can be calculated iteratively using the following equations [43]:

(3) $$i_{t}=\sigma \left(W_{ix}x_{t}+W_{im}m_{t-1}+W_{ic}c_{t-1}+b_{i}\right)$$

(4) $$f_{t}=\sigma \left(W_{fx}x_{t}+W_{fm}m_{t-1}+W_{fc}c_{t-1}+b_{f}\right)$$

(5) $$c_{t}=f_{t}⊙c_{t-1}+i_{t}⊙g\left(W_{cx}x_{t}+W_{cm}m_{t-1}+b_{c}\right)$$

(6) $$o_{t}=\sigma \left(W_{ox}x_{t}+W_{om}m_{t-1}+W_{oc}c_{t}+b_{o}\right)$$

(7) $$m_{t}=o_{t}⊙h\left(c_{t}\right)$$

(8) $$y_{t}=W_{ym}m_{t}+b_{y}$$

**Figure 10:**
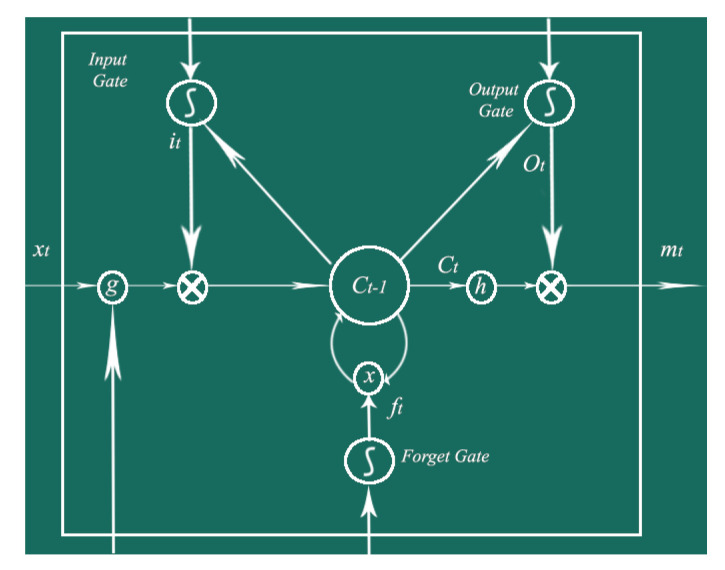
Architecture of a LSTM Memory Unit in Hidden Layers.

In the above equations, σ() represents the sigmoid function, which is defined as:

(9) $$\sigma \left(x\right)=\frac{1}{1+e^{-x}}$$

and the ⊙ in Equations (3)–(8) represents the dot product (also known as scalar product). A memory block, shown in Figure 10, has an input gate, an output gate, and a forget gate. The output of the input gate is represented as $i_{t}$, that of the output gate as $o_{t}$, and the output of the forget gate as $f_{t}$, where $c_{t}$ and $m_{t}$ represent the cell and memory activation vectors, respectively. Similarly, *W* and *b* represent the weight and the bias matrix which are used to establish connections between input layer, memory block, and output layer. g(x) and h(x) are centered logistic sigmoid functions.

### 3.4. Data Normalization

Data normalization is one of the most important steps in data pre-processing. It guarantees the quality of the data before we use as the input to machine/deep learning models [44]. Data normalization is required when features have different ranges of values. For example, in our dataset, the traffic intensity values range approximately between 0 and 1500 while the value ranges for CO and $NO_{2}$ are 0–3.4 and 0–616, respectively. This difference of scale may lead to the poor performance of a machine/deep learning model. Data normalization helps to deal with data that contains values that have different scales. Moreover, it also helps to reduce the training time. Different kind of data normalization techniques are available, including min-max, median normalization, and Z-score decimal scaling. In this paper, we used the most popular normalization technique, min-max normalization [45].

#### Min-Max Normalization

Min-max normalization maps data into pre-defined ranges i.e., [0,1] or [−1,1]. The values of each attribute in the data are defined according to their minimum and maximum value. If we denote the attribute in the data by “Atr”, its value by “a_val”, its normalized value as “a_norm”, and pre-defined range as [lower_lim,higher_lim], then following equation [44] can be used to calculate normalized values between the range [lower_lim,higher_lim]:

(10) $$a\_norm=lower\_lim+\frac{\left(higher\_lim-lower\_lim)\times (a\_valu-min(Atr)\right)}{max(Atr)-min(Atr)}$$

### 3.5. Hyperparameter

We used the following configuration of a LSTM RNN to forecast traffic flow using Madrid’s open data:

- 3 LSTM layers;
- **Dropout:** To keep our model from going into overfitting, we applied dropout [46] at each LSTM layer with a value of 0.7;
- **Early Stopping:** To stop the training before the model approaches overfitting, we used early stopping [47] with the patience value of 5;
- **Look Back Steps:** In order to do prediction at time *t*, “look back” shows how many previous time steps need to be considered. We set the “look back” steps value at 168, which represents the total number of hours in a week. We chose 168 hours (one week) as “look back” period. The plan is to capture the evolution of the air pollutants over a period in which different, but recursive patterns may occur, e.g., working day traffic vs. Week-end traffic. We wanted to grasp the differences between working days and week-end. In addition, in such a period, the pollutants have time to consolidate (some pollutant can float for hours or more). Moreover, this time period could result a better forecasting. Traffic intensity shows different patterns between weekdays and weekends. Pollution “signatures” refer to longer and more complex situations. A week within a particular month (e.g., December before Christmas time) can be characterized by higher volume of traffic and hence pollution. Different months can have very different levels of traffic and pollution. The choice of considering one week is due to the possibility to grasp these variations, while still maintaining a short period for observation and data capture. With respect to pollution, a longer period of time (e.g., a month) would allow a more specific characterization of the traffic in that specific month and the related pollution signature could be used in order to help the prediction. A shorter period of time (one day, two days) is not able to capture these variations in traffic intensity and pollution measurements. However, the choice of one week is a starting point and, for further work, a better tuning of the time could be envisaged.

## 4. Dataset and Performance Evaluation

This section describes the dataset and its features, and evaluates the performance achieved by LSTM RNN for traffic flow forecasting using air pollution and atmospheric data. Open data from Madrid, Spain [37] collected and normalized for 1 year of observations. A large set of data related to traffic intensity was collected in the first step. This dataset also contained weather and pollution-related features. We conducted experiments using the data from two air pollution sensor stations (Figure 4a) to forecast traffic flow. These stations measure $CO,NO,NO_{2},NO_{x}$ and $O_{3}$ values in the air. In addition, we used timestamp, traffic intensity, and atmospheric data, including temperature, pressure, wind speed, and wind direction from nearby weather stations. For a comparison, in the second step, we only used traffic intensity and timestamp values (with no air pollutant or atmospheric parameters) to forecast the traffic flow, and compared the results to see the effect of considering air pollutant and atmospheric data.

We chose 25 traffic flow sensors in a 500 m radius of the two air pollution sensor stations (Figure 4b). Traffic flow data is available after every 15 minutes, however, other data, including CO,NO,$NO_{2},$$NO_{x},$$O_{3},$Pressure,Temperature,WindSpeed, and WindDirection are updated hourly.

As the air pollutant data and atmospheric data are available hourly, therefore, we collected the hourly traffic data to keep it coherent with air pollution and atmospheric data. Table 1 represents the details of the features used to train the model. As our data were organized hourly (from 01 January 2019 to 31 December 2019), we had 8760 records in total; 67% of our data were used for training and 33% were used for testing. In order to extract the traffic flow insights for the roads where sensors are deployed, Table 2 represents the statistics of 25 traffic flow sensors within the chosen distance from the associated air pollution sensor station, and the minimum, maximum, and average traffic flow in the year 2019. Out of 25 sensors, 9 were faulty and gave either null value or garbage values. For those sensors, the minimum, maximum, and average flow values are represented as “NA” in Table 2.

### 4.1. Evaluation Metrics

In order to evaluate the results of the experiments, we defined some metrics to be used for the evaluation of our model. We used two of the most-used evaluation metrics *Mean Absolute Error (MAE)* and *Means Squared Error (MSE)*. Their mathematical representations are [48,49]:

(11) $$MAE=\frac{1}{N}\sum_{i=1}^{N}|y_{i}^{predicted}-y_{i}^{observed}|$$

(12) $$MSE=\frac{1}{N}\sum_{i=1}^{N}{\left(y^{predicted}-y^{observed}\right)}^{2}$$

MAE is not sensitive to outliers. It does not deal well with big errors. It is very useful for continuous variable data. MSE is very useful when the dataset contains outliers. At the beginning of the analysis, we wanted to be sure to grasp insights from very different data and patterns (traffic intensity and air pollutants). For this reason, we decided to check our results using both MSE and MAE. However, in our case, we found out that MAE alone could be used to evaluate the whole performance. Therefore, in future work, for additional experiments, we will use MAE for the evaluation. We used the training loss and the validation loss in the learning curve in order to be sure that our model was not overfitting.

### 4.2. Results

This section provides the MAE and MSE scores of the LSTM RNN model for each of the operational traffic flow sensors (excluding faulty sensors). As explained in the previous section, 25 traffic intensity sensors were considered, and out of those 25, 9 sensors were faulty and so were eliminated from the dataset during the experiments. Hence, Table 3 presents the MAE and MSE scores of 16 traffic flow sensors. We performed an hourly forecast. In order to do that, we determined the traffic intensity at time *t* by considering traffic intensity data, air pollution data, and atmospheric data from [0,t−1] and, air pollution data and atmospheric data from time *t*.

The maximum MAE produced by the LSTM RNN for the traffic sensors within the radius of 500 m of air pollution sensor “28079016” was 0.214 while the minimum MAE was 0.061. Similarly, the maximum MSE was 0.60 and the minimum MSE was 0.009. In order to evaluate our LSTM RNN model further, we conducted the same experiments for air pollution sensor station “28079035” and 5 traffic flow sensors within its 500 m radius. Out of those 5 traffic flow sensors, 3 were faulty. Hence, Table 3 presents the values of 2 of the operational traffic flow sensors (4303 and 10387) around the station “28079035”. The LSTM RNN produced values 0.105 MAE and 0.017 MSE for traffic flow sensor “4303”, and 0.136 MAE and 0.029 MSE for traffic flow sensor “10387”.

**Figure 11:**
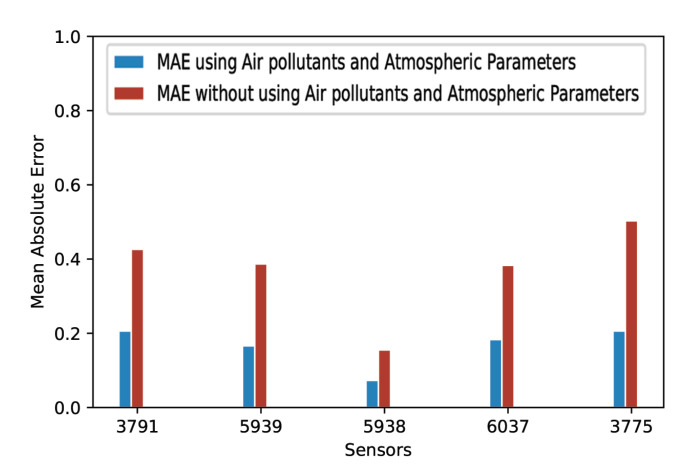
MAE with and without using air pollutants and atmospheric parameters.

**Figure 12:**
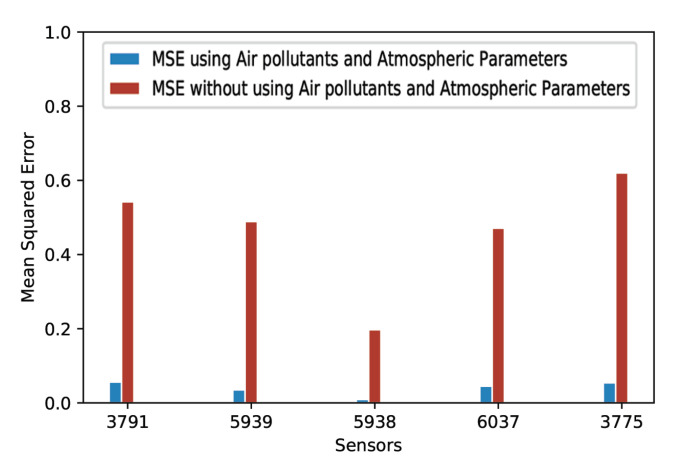
MSE with and without using air pollutants and atmospheric parameters.

In order to observe the effect of introducing air pollutants and atmospheric parameters, we randomly selected five traffic intensity sensors and performed forecasting, considering only timestamped traffic intensity values. Figure 11 and Figure 12 represent the comparative analysis of the mean absolute error and the mean squared error, respectively, with and without using air pollutants and atmospheric parameters as input features. It is clear that air pollutants and atmospheric parameters improve the MAE and the MSE. Our LSTM recurrent neural network-based approach performed better for all of the five considered traffic intensity sensors when air pollutants and atmospheric parameters were used along with the timestamped traffic intensity values.

### 4.3. Further Evaluation

To further evaluate the LSTM RNN model, we determined if our model was overfitting or not. One of the most-widely used methods for verifying overfitting [50,51] is to plot learning curves. A learning curve plots a model’s training loss and validation loss. These curves give information about overfitting and underfitting:

- **Overfitting** represents the ability of the model to learn too much during the training process, so that when unseen data are provided for prediction, it shows poor performance. Overfitting can be diagnosed by plotting learning curves. If the training loss is decreasing but validation loss starts increasing after a specific point, this shows that a model is overfitting [51].
- **Underfitting** represents the inability of the model to learn from training data. If a learning curve shows either of the following two behaviors, the model is underfitting:
  – Validation loss is very high and training loss is flat regardless of training time.
  – Training loss is continuously decreasing without being stable until the training is complete.

**Figure 13:**
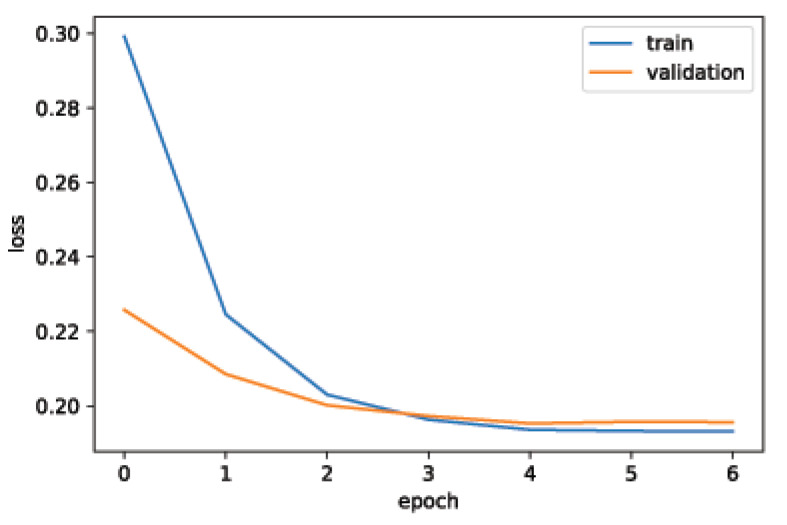
Learning curve representing training and validation losses of the LSTM RNN model for traffic flow forecasting.

Given above definitions, we plotted learning curves to observe the behavior of our model. Figure 13 shows that the learning curve of our model is not following any of the above-mentioned definitions of overfitting and underfitting. Training loss is decreasing and after a specific point it becomes stable. Similarly, validation loss becomes stable and remains close to the training loss. Both of these observations show that our model is a good fit.

### 4.4. Threat to Validity

The model utilized with the currently available data in Madrid. The penetration of electric vehicles may be a factor impacting the generation of pollution in major cities. This could have also a long term impact on our forecasts. However, the substitution of older vehicles with hybrid or electric ones will be relatively quick but not immediate. This delay will give the model some time to adapt and learn the new patterns. Given the ongoing concerns about air pollution, the use of electric vehicles is increasing around the world. For example, the national electric mobility mission plan is anticipating the sale of around 7 million electric vehicles yearly from 2020 onwards [52]. While it will take a long time to completely eliminate conventional vehicles, the elimination of conventional fuel vehicles could be a threat to our approach’s validity, as it is partially dependent upon vehicular pollution emission.

## 5. Conclusions

Traffic forecasting is one of the most important tasks for big cities. Accurate traffic flow forecasting can help drivers to better plan their trips. To provide accurate traffic flow forecasting, this work, first combined air pollutants and atmospheric data with traffic intensity data to forecast traffic flow in Madrid, Spain. In the second step, only timestamped traffic intensity data were used to forecast traffic flow, and then those results were compared with the results from the experiments at step one. The comparison was carried out to observe the effect of adding air pollutants and atmospheric data to forecast the traffic flow. We used a long short-term memory recurrent neural network (LSTM RNN) to perform traffic flow forecasting, with time-series traffic flow, air pollution, and atmospheric data collected from the open datasets of Madrid, Spain. Air pollutants ($CO,NO,NO_{2},NO_{X},$ and $O_{3})$, which are associated with road traffic, were considered as the input features, along with atmospheric variables (wind speed, wind direction, temperature and pressure), because in air pollution dispersion models, these features influence the dispersion of air pollution. Together these features helped the model to better forecast the traffic flow. Experimental results show that addition of air pollutant and atmospheric information with timestamp improved the performance.

### Future Work

In future work, we plan to extend our experiments to assess the effects of seasons, e.g., summer and winter. Traffic patterns are likely to be different in August in Europe, as many people leave cities and go on vacations. Moreover, we want to identify the percentage of air pollution contributed by road traffic and heating/cooling systems in homes, offices, and factories. In addition, we are planning to take air pollution dispersion models like Ausplume and Calpuss into account to better understand the behavior of air pollution. The correlation between air pollution and traffic intensity may differ in different areas of the city. Density of the infrastructure can have an impact on the correlation. In this paper, we only considered two areas in Madrid. However in the future, we plan to take multiple areas and their infrastructure into account to observe the correlation between traffic flow and air pollutants. As a goal, we want to understand if it is possible to analyze the ’signatures’/traces of pollution in order to derive and predict information for correlated phenomena. At the same time, satellite pollution measurements will be taken into consideration in order to understand if they can be used together with ground values to better identify the correlations. In this paper, we considered one of the popular neural network models, i.e., LSTM recurrent neural network. However, some studies [53] show that traditional machine learning models can sometimes perform better than deep learning techniques. In addition to traditional machine learning models, statistical models have also been found to perform better than machine learning models [54]. Hence, it is an open research question to choose the better machine/deep learning model combined with air pollution and atmospheric data.

In addition, we want to investigate how to optimize the fusion of different sources of information to improve the prediction for relevant phenomena in the cities. The deployment and maintenance of a large sensor network for traffic and air quality monitoring is a large investment that requires careful planning in order to be effective and practical. There are a few cities (Madrid is one), that have similar deployment and provide open access to data [37,55,56]. Many other cities cannot afford such an investment. This means that monitoring may be very active in certain areas while areas nearby are not similarly controlled. We will work on pollution data analysis to verify if it is possible to adequately monitor pollution and to derive and predict phenomena related/associated to it. Another aspect that will be further studied is the possibility offered by the fusion of data in reducing the number of sensors in a city without lowering the information quality, which will ultimately lead to a reduction in cost. For instance, in Madrid, some traffic sensors could be eliminated in favor of more air control sensors if a strong relationship can be verified between traffic and pollution levels.

## Figures and Tables

**Table 1:** Features used for training the model.

Feature	Value/Unit
Month	1–12
Day	1–28/29/30/31
Weekday	1–7
Hour	0–23
CO	mg/m${}^{3}$
NO	μg/m${}^{3}$
$NO_{2}$	μg/m${}^{3}$
$NO_{x}$	μg/m${}^{3}$
$O_{3}$	μg/m${}^{3}$
Pressure	mb
Temperature	${}^{\circ }C$
Wind Direction	Angle
Wind Speed	m/s
Traffic Flow	Vehicles/h

**Table 2:** Traffic flow sensors’ statistics.

Air Pollution Sensor Station	Traffic Flow Sensor	Distance from Air Pollution Sensor Station	Minimum Flow (Annual)	Maximum Flow (Annual)	Average Flow (Annual)
28079016	6037	240 m	0	384	112.344
	3791	79 m	4	1601	493.693
	3775	294 m	17	1166	468.615
	5938	205 m	0	220	32.011
	5939	125 m	5	1980	522.943
	10124	242 m	NA	NA	NA
	6058	214 m	NA	NA	NA
	3594	296 m	NA	NA	NA
	5922	366 m	NA	NA	NA
	10128	500 m	4	1413	437.701
	10125	455 m	NA	NA	NA
	5941	303 m	0	1324	135.017
	5923	426 m	5	1334	437.864
	5994	483 m	0	480	135.389
	5940	369 m	NA	NA	NA
	5942	336 m	0	1523	534.091
	5944	349 m	0	182	72.176
	5921	374 m	23	1214	481.669
	3776	425 m	17	1208	476.911
	5937	484 m	0	313	86.216
28079035	3731	26 m	NA	NA	NA
	4303	39 m	0	181	52.188
	3730	133 m	NA	NA	NA
	4301	137 m	NA	NA	NA
	10387	196 m	40	1260	608.482

**Table 3:** Mean absolute error (MAE) and mean squared error (MSE) for two considered traffic flow forecasting for considered traffic flow sensors.

Air Pollution Sensor Station	Traffic Flow Sensor	MAE	MSE
28079016	6037	0.183	0.045
	3791	0.206	0.056
	3775	0.206	0.054
	5938	0.073	0.009
	5939	0.166	0.035
	10128	0.203	0.053
	5941	0.061	0.005
	5923	0.188	0.046
	5994	0.173	0.047
	5942	0.214	0.060
	5944	0.208	0.056
	5921	0.200	0.051
	3776	0.193	0.051
	5937	0.160	0.030
28079035	4303	0.105	0.017
	10387	0.136	0.029
